# The challenges of recruiting cancer patient/caregiver dyads: informing randomized controlled trials

**DOI:** 10.1186/s12874-018-0614-7

**Published:** 2018-11-21

**Authors:** Leila Heckel, Kate M. Gunn, Patricia M. Livingston

**Affiliations:** 1 Faculty of Health, School of Nursing and Midwifery, Deakin University, Geelong, Victoria 3220 Australia; 2 Cancer Council SA, 202 Greenhill Road, Eastwood, South Australia 5063 Australia; 3 Flinders Centre for Innovation in Cancer, School of Medicine, Flinders University, Sturt Road, Bedford Park, South Australia 5042 Australia; 4 University of South Australia Cancer Research Institute, North Terrace, Adelaide, 5000 South Australia; 5 Faculty of Health, Deakin University, Geelong, Victoria 3220 Australia

**Keywords:** Participation rates, Recruitment, Randomized controlled trials, Caregivers, Dyads, Cancer, Oncology, Telephone intervention

## Abstract

**Background:**

Family members are increasingly involved in the care of cancer patients, however many are not prepared for this challenging role. Intervention-based studies are valuable to inform the most appropriate and effective support for caregivers. Barriers in the recruitment of patient/caregiver dyads exist but the reasons for non-participation are less well understood. This analysis determined the factors associated with participation in a randomized controlled trial involving patient/caregiver dyads, reasons for non-participation and factors associated with these reasons.

**Methods:**

Patients with any type of cancer (other than non-melanoma skin cancer), and their caregiver were recruited at four Australian health services. Eligible patients were invited to participate together with their caregiver (*N* = 737). Non-participation data were collected from non-participants. Bivariate and binary logistic regression analyses were conducted to examine factors associated with participation.

**Results:**

Of the 737 eligible dyads, 521 (71%) declined participation. Dyad characteristics associated with participation were caregiver gender, patient treatment modality and hospital type. The odds for participating were almost two times greater for female than male caregivers (*p* = 0.005); 13 times greater for patients receiving chemoradiotherapy compared to radiotherapy alone (*p* < 0.001); and three times greater for dyads attending a private versus public hospital (*p* < 0.001). Reasons for non-participation were lack of interest (33%), lack of time (29%), not requiring support (23%), too burdensome (15%); factors significantly associated with these reasons were treatment modality, patient age, cancer type, and hospital type. Patients diagnosed with prostate cancer and receiving chemotherapy alone were less likely to decline due to a lack of interest. Patients more likely to decline due to lack of time were those aged 40–59 years and receiving chemotherapy alone. Patients who were more likely to decline because they felt participation was too burdensome were those attending a private hospital for treatment.

**Conclusions:**

To optimize recruitment, it is recommended that special attention is given to different cancer types and treatment modalities, gender and age. Approaching dyads at varied time points when their need for support is high is recommended. This analysis provides important information for researchers undertaking randomized controlled trials involving people diagnosed with cancer and their caregivers.

## Background

Involvement of family members and friends in the provision of care for people diagnosed with cancer in the home setting has become common place worldwide [1]. In many instances, informal caregivers take on caregiving responsibilities with little or no experience or support [2], resulting in increased caregiver burden and poorer patient outcome [3–5]. Supporting family members in their role as caregivers is critical and can be achieved through intervention-based studies, which can vary in design and approach. Randomized controlled trials represent a good experimental design but testing the efficacy of such interventions requires adequate numbers of trial participants [6], and recruitment of cancer dyads into clinical research is challenging with accrual rates ranging from 5 to 50% [6–11]. Recruitment for couple-based interventions requiring *both* the patient *and* the caregiver to be involved in the intervention at the same time, is known to be particularly difficult [6, 12–14].

When embarking on recruiting cancer dyads, understanding the barriers and facilitators is important. Identifying and accessing caregivers to participate in studies can be difficult due to caregivers’ time constraints and/or own health issues [9, 11]. Further, demographic characteristics, such as being male [7, 10, 14], being older [15], and some clinical factors (e.g. cancer diagnosis other than breast and prostate cancer, advanced disease) [14], have been found to be associated with lower patient accrual rates.

Establishing a profile of non-participants helps identify those who are less likely to participate in research and provides opportunities to proactively address these matters when designing research studies. However, an analysis of participation results is not often reported in the literature [15].

The aim of this analysis was to examine the characteristics associated with accrual into a multi-centred, randomized controlled trial involving caregivers of cancer patients, receiving treatment with curative intent.

## Methods

### Study design

**Fig. 1:**
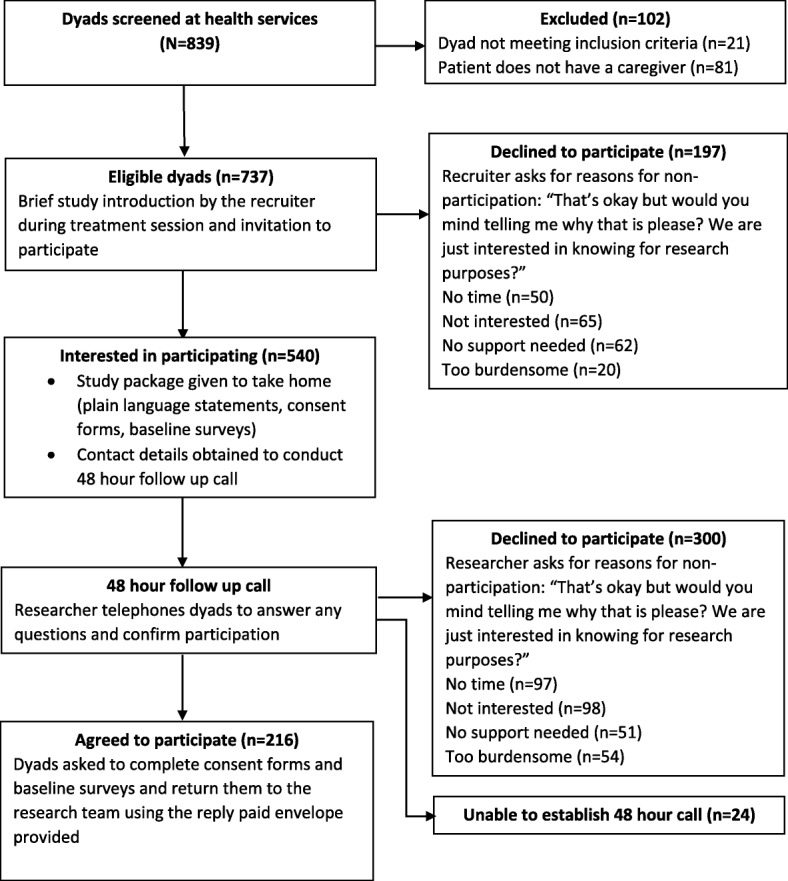
Flowchart outlining the recruitment and consent process of the PROTECT study

From August 2013 to December 2014, patient/caregiver dyads were recruited at three Australian public health services and one private health service. Potentially-eligible patients were identified by trained senior nurses using treatment lists, at each participating health service. Those newly diagnosed with a primary cancer (approximately 2-months post-diagnosis), attending cycles 2–5 of adjuvant chemotherapy and/or fraction 2–10 for radiotherapy and receiving treatment with curative intent, were eligible to participate. From our pilot work, we determined this was the optimal timeframe in which to approach prospective participants, however, it should be noted that many patients received more than five cycles of chemotherapy/10 fractions of radiation treatment following enrolment into the trial. Each patient and his/her caregiver were then approached by a trained researcher during their scheduled treatment visit and further eligibility checks were conducted (English proficiency, no cognitive dysfunction, patient has a caregiver, patient and nominated caregiver both > 18 years). Eligible dyads were given a brief introduction and a study package containing consent forms, information sheets and baseline surveys. If the dyad was interested in the study, initial consent was sought for a researcher to re-contact them within 48 h to give them time to discuss their participation in private rather than during their treatment session. If the caregiver was not present at the treatment-related appointment, consent was sought from the patient to provide contact details of the caregiver, so that the researcher could conduct the 48 h follow up call. Interested dyads were then telephoned to answer any questions and to confirm participation. Consenting dyads were asked to complete the consent form and baseline survey, and return them to the researchers using the reply paid envelope provided. If the dyad was unavailable at the time of calling, up to seven call attempts were made to contact the dyad to seek confirmation of participation. Non-participation occurred if the dyad or either one of them declined participation, or after seven unsuccessful attempts to contact them. For non-consenting dyads, the reason for refusal was documented (one main reason per dyad only as it was not deemed appropriate to burden them with too many questions given their desire not to participate). Interested dyads were asked to complete the consent form and baseline questionnaires (patient and caregiver survey) and post them back to the researchers using a reply-paid envelope. Figure 1 outlines the recruitment and consent process of the PROTECT study. Consenting dyads were then randomized into the telephone-based intervention group or the telephone-based attention control group [16]. Participants in both groups received three telephone calls; one at the beginning of the program, as well as one month and four months later. While the intervention group received a tailored intervention program delivered by both the Victorian and South Australian based Cancer Council telephone information and support services/helplines (known as ‘Cancer Council 13 11 20’, which is the largest non-government provider of cancer support services in Australia), caregivers in the attention control group received a sham intervention with a researcher providing them with the contact details of the Cancer Council telephone information and support service, which they could contact if needed. If they chose to call that service, they received the usual 13 11 20 telephone support not the tailored intervention. In addition to the baseline survey, caregivers were asked to complete questionnaires one month and six months post-intervention, and patients completed questionnaires one month post-intervention. Patient data were collected to assess the potential impact of the caregiver intervention on patient outcomes. A more complete description of the study and findings on the efficacy of the intervention on reducing caregiver burden are described elsewhere [17].

Ethics approval was obtained from Deakin University Human Research Ethics Committee: 2012–083, the respective committees at each participating health service and the two Cancer Councils. The study was registered with the Australian New Zealand Clinical Trial Registry: ACTRN12613000731796.

### Data collection and analyses

Patient information was obtained through medical records and included: age, gender, postcode, cancer diagnosis, treatment type (chemotherapy alone, radiotherapy alone, chemoradiotherapy), and hospital type (public, private). Caregiver information was collected from consenting dyads through the baseline questionnaire and included, age, gender, type of relationship to patient (spouse/partner, parent, adult child, other relative, friend/other), whether or not they resided with the patient (yes/no), household size and level of education (secondary school, diploma/certificate, university degree). Caregiver data from non-participating dyads was restricted to caregiver gender. The socioeconomic status (SES) of participants was measured by assigning the Socio-Economic Indices for Areas (SEIFA) decile rankings (1–10) to the postcode [18]; a method developed by the Australian Bureau of Statistics which has been widely used in research studies [19, 20]. To represent relative advantage/disadvantage, rankings were grouped into the following categories: low SES (1–3), middle SES (4–7), and high SES (8–10). Reasons for decline were grouped into the following categories: lack of time, lack of interest, not requiring support, too burdensome (questionnaires/study period too long, caregiver and/or patient too unwell). We were unable to collect reasons from those dyads who could not be contacted for the 48 h follow-up call (*n* = 24). All types of cancers other than non-melanoma skin cancer were included and these were grouped as follows: breast, bowel, prostate/testicular, haematological (e.g. leukemia, lymphomas), lung/ head and neck/ brain/ melanomas, and others (e.g. gynecological, bladder, kidney, soft tissue).

The data were analysed using Statistical Package for Social Sciences (SPSS), version 21. Descriptive statistics were performed to describe sample characteristics. Bivariate analyses were performed to compare participants and non-participants on demographic variables including caregiver gender, patient age and gender, socio-economic status (SES), cancer diagnosis, treatment type and hospital type. Associations between these variables and the reasons for non-participation were also examined. Variables which produced significant results in bivariate analyses were then included in the binary logistic regression (multivariable analysis) to ascertain the effects of caregiver and patient factors on the likelihood that dyads participated in the study. Results of the analyses were considered statistically significant when *p* < 0.05.

## Results

A total of 839 patients were screened, 102 (12%) were excluded as either the patient or the caregiver did not meet eligibility criteria including 81 patients (10%) who did not have a caregiver (Fig. 1). From a total of 737 eligible patient/caregiver dyads, 521 (71%) declined participation. Reasons stated by dyads for non-participation were ‘lack of interest’ (*n* = 163, 33%), ‘lack of time’ (*n* = 147, 29%), ‘not requiring support’ (*n* = 113, 23%), and ‘too burdensome’ (*n* = 74, 15%).

### Profile of participants and non-participants

The profile of participants and non-participants are presented in Table 1.

**Table 1:** Profile of non-participants and participants, and factors associated with participation (*N* = 737)

		Non-Participants *n* = 521 (71%)	Participants *n* = 216 (29%)	*P value*
Caregiver				
Gender^a^	Male^b^	218 (70)	92 (30)	0.012
	Female	187 (60)	124 (40)	
Age	Mean (SD)		56.75 (12.94)	–
	18–39 years		23 (11)	
	40–59 years		97 (45)	
	60+ years		96 (44)	
Living situation	Together		181 (84)	–
	Not together		35 (16)	
Patient/caregiver Relationship	Spouse/partner		171 (79)	–
	Non-spouse/partner		45 (21)	
Education^a^	Tertiary		68 (32)	–
	Non-tertiary		147 (68)	
Household size	Median		2.00	–
	Min/Max (Range)		1/8 (7)	
Patient				
Gender	Female^b^	332 (73)	122 (27)	0.057
	Male	189 (67)	94 (33)	
Age	Mean (SD)	59.96 (12.56)	56.29 (12.25)	
	60+ years^b^	284 (72)	112 (28)	
	40–59 years	207 (70)	90 (30)	0.592
	18–39 years	30 (68)	14 (32)	0.463
Socio-economic status^a^	Middle^b^	187 (68)	88 (32)	
	Low	148 (76)	47 (24)	0.063
	High	185 (70)	79 (30)	0.602
Cancer type	Breast^b^	240 (73)	89 (27)	
	Bowel	40 (69)	18 (31)	0.722
	Prostate/testicular	59 (67)	29 (33)	0.276
	Lung/H&N/melanoma/brain	78 (70)	33 (30)	0.659
	Haematological	55 (68)	26 (32)	0.438
	Others	49 (70)	21 (30)	0.431
Treatment type	Radiotherapy^b^	312 (81)	72 (19)	
	Chemotherapy	189 (70)	82 (30)	0.003
	Chemoradiotherapy	20 (24)	62 (76)	< 0.001
Hospital type	Public^b^	448 (76)	144 (24)	< 0.001
	Private	73 (50)	72 (50)	

^a^n varies due to missing data, ^b^reference category

#### Non-participant profile

Caregiver data available for those dyads who declined participation were limited to gender and there were slightly more male (54%) than female caregivers in this group. Non-participating patients’ mean age was 59.96 (SD: 12.56), 64% of patients were female and most of them came from middle (36%) and high (35%) socio-economic areas. Forty-six percent were diagnosed with breast cancer, 11% with prostate/testicular cancer, and 11% with a haematological cancer. The majority (86%) attended a public hospital for treatment and 60% were receiving radiotherapy alone.

#### Participant profile

Of the participating dyads, caregiver mean age was 56.75 (SD: 12.94), more than half (57%) were female caregivers and 32% percent had a tertiary education. The majority (79%) were in a spousal/partner relationship with the person they provided care for, and 84% lived in the same household. Patient mean age was 56.29 (SD: 12.25), 56% of patients were female and most of them came from middle (41%) and high (37%) socio-economic backgrounds. Forty-one percent were diagnosed with breast cancer, 14% with prostate/testicular cancer, and 12% with a haematological cancer. Two-thirds (67%) attended a public hospital for treatment and 33% were receiving radiotherapy alone.

### Factors associated with participation

Bivariate analysis revealed participation was associated with caregivers being female (*p* = 0.012), patients receiving chemotherapy alone (*p* = 0.003), chemoradiotherapy (*p* < 0.001), and being treated in a private hospital (*p* < 0.001) (Table 1). In the multivariable analysis, these variables remained significantly associated with participation (Table 2). Dyads where the caregiver was female were almost twice as likely to participate compared to those where the caregiver was male (OR = 1.69 [95% CI, 1.2 to 2.4], *p* = 0.005); patients receiving chemoradiotherapy increased the odds of participating in the study by almost 13 times compared to receiving radiotherapy alone (OR = 12.91 [95% CI, 6.9 to 24.1], *p* < 0.001); and the odds of participation were three times greater for dyads attending a private hospital for treatment than for those attending a public hospital (OR = 3.08 [95% CI, 2.0 to 4.7], *p* < 0.001).

**Table 2:** Binary logistic regression analyses for factors associated with participation and reasons for non-participation

					Unadjusted	95% CI		
		B	S.E.	Wald χ^2^	*OR*	Lower	Upper	*p*-value
Participating in the study								
Intercept		− 1.7	0.2	81.4	0.2			< 0.001
Carer gender	Female	0.5	0.2	7.7	1.7	1.2	2.4	0.005
Treatment type (reference = radiotherapy)	Chemotherapy	0.3	0.2	3.1	1.4	1.0	2.1	0.080
	Chemoradiotherapy	2.6	0.3	64.2	12.9	6.9	24.1	< 0.001
Hospital type	Private	1.1	0.2	27.6	3.1	2.0	4.7	< 0.001
Lack of time								
Intercept		−1.3	0.2	38.2	0.3			< 0.001
Patient age (reference = 60–90 years)	18–39 years	0.7	0.4	2.5	1.9	0.9	4.3	0.113
	40–59 years	0.4	0.2	3.9	1.6	1.0	2.4	0.049
Patient gender	Male	−0.2	0.3	0.4	0.8	0.4	1.5	0.529
Patient diagnosis (reference = breast cancer)	Others^a^	− 0.5	0.4	1.2	0.6	0.3	1.4	0.271
	Bowel	−0.3	0.4	0.7	0.7	0.3	1.6	0.395
	Prostate	−0.0	0.5	0.0	1.0	0.4	2.5	0.938
	Lung^b^	−0.3	0.4	0.5	0.8	0.4	1.6	0.496
	Haematological	0.0	0.4	0.0	1.0	0.5	2.2	0.982
Treatment type (reference = radiotherapy)	Chemotherapy	0.6	0.2	6.9	1.9	1.2	3.0	0.009
	Chemoradiotherapy	0.4	0.5	0.7	1.5	0.5	4.3	0.417
Hospital type	Private	0.3	0.3	1.2	1.36	0.8	2.4	0.277
Lack of interest								
Intercept		−0.3	0.2	2.9	0.7			0.091
Patient age (reference = 60–90 years)	18–39 years	−0.3	0.4	0.5	0.7	0.3	1.8	0.483
	40–59 years	−0.4	0.2	3.5	0.7	0.4	1.0	0.060
Patient diagnosis (reference = breast cancer)	Others	0.6	0.3	2.8	1.8	0.9	3.5	0.097
	Bowel	0.1	0.4	0.0	1.1	0.5	2.3	0.855
	Prostate	−1.1	0.4	8.4	0.3	0.1	0.7	0.004
	Lung	−0.2	0.3	0.3	0.8	0.5	1.5	0.551
	Haematological	0.4	0.4	1.4	1.5	0.8	3.1	0.232
Treatment type (reference = radiotherapy)	Chemotherapy	−0.6	0.2	6.4	0.5	0.3	0.9	0.012
	Chemoradiotherapy	0.1	0.5	0.0	1.1	0.4	2.9	0.872
Hospital type	Private	−0.4	0.3	1.8	0.6	0.3	1.2	0.177
Not requiring support								
Intercept		−1.0	0.2	20.8	0.4			< 0.001
Patient age (reference = 60–90 years)	18–39 years	−1.3	0.8	2.7	0.3	0.1	1.3	0.100
	40–59 years	−0.3	0.2	1.2	0.8	0.5	1.2	0.270
Patient diagnosis (reference = breast cancer)	Others	−1.2	0.6	3.7	0.3	0.1	1.0	0.053
	Bowel	0.4	0.4	1.0	1.5	0.7	3.3	0.322
	Prostate	0.8	0.3	6.2	2.3	1.2	4.3	0.013
	Lung	−0.3	0.3	0.7	0.7	0.4	1.4	0.390
	Haematological	0.0	0.4	0.0	1.0	0.4	2.5	0.928
Treatment type (reference = radiotherapy)	Chemotherapy	−0.6	0.3	4.1	0.5	0.3	1.0	0.042
	Chemoradiotherapy	−1.5	1.0	2.1	0.2	0.0	1.7	0.150
Too burdensome								
Intercept		−2.3	0.2	96.4	0.1			< 0.001
Patient diagnosis (reference = breast cancer)	Others	1.2	0.4	8.7	3.3	1.5	7.3	0.003
	Bowel	0.4	0.5	0.8	1.6	0.6	4.1	0.367
	Prostate	−0.1	0.5	0.0	0.9	0.4	2.4	0.905
	Lung	1.0	0.3	8.1	2.7	1.4	5.3	0.004
	Haematological	−0.1	0.5	0.0	0.9	0.3	2.5	0.858
Hospital type	Private	0.8	0.3	6.3	2.3	1.2	4.5	0.012

^a^includes gynecological, bladder, kidney, soft tissue; ^b^includes lung, head and neck, brain, melanoma

### Factors associated with reasons for non-participation

#### Reason ‘lack of time’

Bivariate analysis showed that dyads were more likely to decline participation due to ‘lack of time’ if the patient was younger (18–39 years, *p* = 0.009; 40–59 years, *p* = 0.001), received chemotherapy alone (*p* < 0.001), and was treated at a private hospital (*p* = 0.019) but less likely to decline if the patient was male (*p* = 0.039) and diagnosed with lung, head and neck, melanoma, or brain cancer (*p* = 0.03). In multivariable analysis, patient’s age and treatment modality remained significantly associated with declining participation (Table 2). Dyads, where the patient was aged 40–59 years, were 1.5 times more likely to decline participation due to a lack of time compared to those aged 60+ years (OR = 1.56 [95% CI, 1.0 to 2.4], *p* = 0.049), and patients receiving chemotherapy alone were nearly twice as likely to decline participation due to a lack of time compared to patients receiving radiotherapy alone (OR = 1.89 [95% CI, 1.2 to 3.0], *p* = 0.009).

#### Reason ‘lack of interest’

Bivariate analysis showed that dyads were less likely to decline participation due to ‘lack of interest’ if the patient was younger (40–59 years, *p* = 0.039), had a diagnosis of prostate cancer (*p* = 0.037), received chemotherapy alone (*p* = 0.031), and was treated at a private hospital (*p* = 0.019). In multivariable analysis, cancer diagnosis and treatment modality remained significantly associated with declining participation (Table 2). Dyads where the patient was diagnosed with prostate cancer compared to those diagnosed with breast cancer (OR = 0.32 [95% CI, 0.1 to 0.7], *p* = 0.004), and dyads where the patient was receiving chemotherapy alone compared to those receiving radiotherapy alone (OR = 0.530 [95% CI, 0.3 to 0.9], *p* = 0.012) were less likely to decline participation due to lack of interest.

#### Reason ‘not requiring support’

Bivariate analysis showed that dyads were more likely to decline participation due to ‘not requiring support’ if the patient was diagnosed with prostate cancer (*p* < 0.001) and less likely to decline if the patient was younger (18–39 years, *p* = 0.030; 40–59 years, *p* = 0.018), diagnosed with a cancer listed as ‘other’ (e.g. gynaecological cancers, bladder; *p* = 0.039), and was receiving chemotherapy alone (*p* = 0.001). In multivariable analysis, patient’s diagnosis and treatment modality remained significantly associated with participation (Table 2). The odds of declining participation because the dyad felt they needed no support were two times greater if the patient was diagnosed with prostate cancer (OR = 2.28 [95% CI, 1.2 to 4.3], *p* = 0.013) than for those diagnosed with breast cancer. Dyads where the patient was receiving chemotherapy alone were less likely to decline due to not requiring support compared to those receiving radiotherapy alone (OR = 0.55 [95% CI, 0.3 to 1.0], *p* = 0.042).

#### Reason ‘too burdensome’

Bivariate analysis showed that dyads were more likely to decline because they felt participation was too burdensome if the patient was diagnosed with lung, head and neck, melanoma, or brain cancer (*p* = 0.014), had a cancer listed as ‘other’ (*p* = 0.008), or was receiving treatment at a private hospital (*p* = 0.044). These variables remained significantly associated with declining participation in multivariable analysis (Table 2). The odds of declining because the dyad felt participation would be too burdensome were three times greater for dyads where the patient was diagnosed with a cancer listed as ‘other’ (OR = 3.29 [95% CI, 1.5 to 7.3], *p* = 0.003) and almost three times greater for those diagnosed with lung, head and neck, melanoma, or brain cancer (OR = 2.69 [95% CI, 1.4 to 5.3], *p* = 0.004) as opposed to those with breast cancer. Dyads attending a private hospital for treatment were twice as likely to decline participation reporting it would be too burdensome compared to those attending a public hospital (OR = 2.32, 95% CI, 1.2 to 4.5], *p* = 0.012).

## Discussion

This analysis reported on the factors associated with participation in a multi-centred randomized controlled trial. We have provided additional informative data on the reasons for non-participation and dyadic factors associated with those reasons, to inform the successful recruitment of cancer dyads into intervention studies in the future. Overall, 216 dyads consented from a total of 737 eligible patient/caregiver pairs, reflecting an uptake rate of 29% which was lower than some randomized controlled intervention studies involving people diagnosed with cancer and their caregivers [7, 9, 12] but higher than others [6, 21, 22]. Of the 102 dyads who did not meet the selection criteria, nearly 80% reported not having a caregiver, highlighting the need to investigate the supportive care needs of people diagnosed with cancer who do not have caregivers- a potentially vulnerable group.

Caregiver gender was identified as a key factor associated with participation. Female caregivers were more likely to participate in our study which is in line with the literature [7, 15] and may be linked to the fact that men are less likely to seek help than females, due to fears about help-seeking being perceived as a sign of weakness or as a lack of emotional control [23].

Patient related factors which influenced participation were treatment modality and hospital type. We found that the odds of participation in the study were 13 times greater if the patient received chemoradiotherapy as opposed to radiotherapy alone. Receiving both regimes can cause a range of side effects exceeding those from radiotherapy alone [24]. It is possible that adverse effects from dual treatments inflicted considerable discomfort to patients and may have required more intense caregiver involvement in the management of side effects, thereby creating a greater need for caregiver support and a greater willingness to participate in our study. This outcome is in line with a qualitative study conducted by Ream et al. 2013 [25] where caregivers of patients undergoing chemotherapy reported a lack of support to address their information and health care needs, resulting in isolation and feelings of insecurity in responding to patients’ needs.

We achieved a higher participation rate at private compared to public hospitals. There is some evidence in the literature suggesting that caregivers of people diagnosed with cancer recruited at private hospitals present with more unmet supportive care needs compared to those from public hospitals [26]. Therefore, higher enrolment of dyads at private health services may reflect their greater need for support. Public hospitals provide high quality medical care in Australia and are more widely accessible, free of charge and are better equipped to deal with more complex cases. On the other hand, private hospitals offer more choice related to patient care (e.g. choice of doctor) and waiting times for elective surgery can be shorter; but to access this, people must have private health insurance.

The most common reasons stated by dyads declining participation were lack of interest, lack of time, not requiring support, and too burdensome, and these reasons were linked to certain patient factors such as treatment modality, age, cancer type, and hospital type. While the majority of dyads preferred not to elaborate on the specific reasons for declining, some stated that they were already receiving support from family members or from a psychologist.

Dyads, where the person with cancer was receiving chemotherapy alone, as opposed to radiotherapy alone, were more likely to decline because they reported they had no time available to participate in our study. However, they were less likely to decline due to lack of interest or because they felt they did not need any support. Compared to the side effects from radiation treatment, those from chemotherapy can be more intense [27] and may cause greater interference with the dyad’s day to day functioning, impacting on the time dyads had available to participate in research. On the other hand, while chemotherapy side effects mainly occur during active treatment and gradually disappear after treatment has ceased, secondary effects from radiotherapy may not be present until several months after completing treatment [28, 29]. In addition, patients receiving radiotherapy attended the radiation department usually on a daily basis; being followed up and supported regularly may minimise the need for further support at this phase of the treatment. Further research is required to better understand why this subgroup was less likely to participate in the study.

We also found that dyads, where the patient was younger (< 60 years), were more likely to decline, reporting they were too busy and did not have the time to take part in the study. Therefore, an intervention that takes into account the time constraints of younger dyads requires investigation. Further research into the acceptability and efficacy of web or app-based interventions to support people diagnosed with cancer and their caregivers is worth pursuing, as these modes of delivery may offer greater flexibility in terms of the times at which they could engage, and thereby be more suitable for younger cancer dyads.

In terms of cancer type, compared to dyads where the patient had breast cancer, those with lung, head and neck, brain, and melanoma were more likely to decline, reporting participation would be too burdensome. Our findings are in line with previous research suggesting lower participation rates in patients with a diagnosis other than breast cancer [14]. The existence of an extensive network of breast care nurses, and the widely held perception that women with breast cancer and their families are better supported than those with other diagnoses, may explain why dyads with other cancers in our study, who may have received little support since diagnosis and were coping less well, perceived research participation as too burdensome. Further, it should be noted that these cancers are often aggressive and treatments often have debilitating side effects which can place heavy burden on caregivers, and together with the perceived burden of participating in research, it may have been particularly difficult for them to take part in the study albeit they could have benefited from the intervention. However, more research is needed to support this argument. Further, dyads where the patient was diagnosed with prostate cancer were more likely to decline participation, reporting they did not need any support but were less likely to decline due to lack of interest. The majority of prostate cancer patients in our study were receiving radiotherapy only (94%) and as mentioned previously this treatment modality is usually associated with fewer side effects during active treatment. It is possible that these dyads were coping well and therefore did not feel the need for any support. In addition, as indicated above, research suggests different help seeking behaviours in men with a greater reluctance to ask for support compared to women, which may be associated with the lower accrual rates for prostate cancer patients found in our study [30].

There are limitations to this study. Caregiver data available for non-participating dyads were restricted to caregiver gender, which limited a comparison of caregivers that enrolled and declined participation. While it was possible to collect information on caregiver gender through observation during the recruitment process at each health service, caregiver demographics were collected through the baseline survey, which was completed by enrolled participants only. The difficulty experienced recruiting dyads into this study was in part due to the need to obtain consent from both the person diagnosed with cancer and the caregiver; we observed one party agreeing to participate while the other chose to decline. In addition, when recording the reasons for non-participation, it was often difficult to specify who (the person diagnosed with cancer or the caregiver) was responsible for declining enrolment. This was particularly the case during the 48 h follow up calls as dyads had time to discuss their potential participation, conversations were usually brief and did not allow for a detailed investigation in to who made the final decision to decline. Further, we did not collect reasons or motivations of those dyads that chose to participate in our trial. Future research should consider obtaining this important information, to improve recruitment of cancer-affected dyads in the future.

## Conclusions

This analysis provides important information for researchers undertaking randomized controlled trials involving people diagnosed with cancer and their caregivers that should be relevant to many intervention and epidemiological studies. We provide new insights into factors associated with enrolling cancer dyads and identified those who are prone to decline participation. Male caregivers were less likely to participate in our study, and those dyads where the patient was receiving a combination of chemotherapy and radiotherapy and being treated at a private hospital, were more likely to take part. Common reasons for declining participation were lack of interest, lack of time, not requiring support, and finding participation too burdensome. Patient factors associated with these reasons were treatment modality, cancer type, age, and hospital type. These results may also be useful when recruiting dyads with other chronic illnesses. A focus on gender specific approaches would be advantageous to increase participation of male caregivers as they are less likely to take part in research than females. In addition, developing programs which are more aligned to the busy time schedules of younger dyads, may help overcome recruitment barriers and more importantly, address unmet needs in this population. Further consideration should be given to the appropriate timing when recruiting dyads, taking into account variations related to cancer type and treatment modality; offering participation when the need for support is highest (e.g. via screening for distress and triaging to support services accordingly). It would be advantageous to explore novel approaches such as technology-based interventions (e.g. smartphone applications) to assist caregivers and patients throughout their cancer trajectory, as this mode of delivery may be perceived as less burdensome and time consuming than a telephone-based intervention. When designing intervention studies for cancer dyads it is also important to consider the various roles caregivers play in relation to the situation of the cancer patient. For example, caregiver tasks can vary with the type of cancer diagnosis as well as with the changing needs of the patient throughout the cancer trajectory. In addition, further research into the characteristics of private and public healthcare environments may help explain differences in participation rates among these health services.

## Abbreviations

ACTRN: Australian Clinical Trial Registration Number
CI: Confidence Interval
H&N: Head and Neck
OR: Odd Ratio
RCT: Randomized Controlled Trial
SD: Standard Deviation
SEIFA: Socio-Economic Indices for Areas
SES: Socio Economic Status
SPSS: Statistical Package for Social Sciences
